# Injectable Lipid-Based Depot Formulations: Where Do We Stand?

**DOI:** 10.3390/pharmaceutics12060567

**Published:** 2020-06-19

**Authors:** Lisa Rahnfeld, Paola Luciani

**Affiliations:** Pharmaceutical Technology Research Group, Department of Chemistry and Biochemistry, University of Bern, Freiestrasse 3, 3012 Bern, Switzerland; lisa.rahnfeld@dcb.unibe.ch

**Keywords:** injectable lipid depot, long-acting drug delivery system, oil-based solutions, liposomes, in situ forming systems, implants, solid particles

## Abstract

The remarkable number of new molecular entities approved per year as parenteral drugs, such as biologics and complex active pharmaceutical ingredients, calls for innovative and tunable drug delivery systems. Besides making these classes of drugs available in the body, injectable depot formulations offer the unique advantage in the parenteral world of reducing the number of required injections, thus increasing effectiveness as well as patient compliance. To date, a plethora of excipients has been proposed to formulate depot systems, and among those, lipids stand out due to their unique biocompatibility properties and safety profile. Looking at the several long-acting drug delivery systems based on lipids designed so far, a legitimate question may arise: How far away are we from an ideal depot formulation? Here, we review sustained release lipid-based platforms developed in the last 5 years, namely oil-based solutions, liposomal systems, in situ forming systems, solid particles, and implants, and we critically discuss the requirements for an ideal depot formulation with respect to the used excipients, biocompatibility, and the challenges presented by the manufacturing process. Finally, we delve into lights and shadows originating from the current setups of in vitro release assays developed with the aim of assessing the translational potential of depot injectables.

## 1. Introduction

Medication non-compliance is a dreadful bottleneck for successful treatment outcomes in a multitude of diseases. Among the factors threatening patient adherence, a high number of daily doses, the duration of the condition (acute versus chronic), and the transition to chronification as well as adverse side effects pose severe challenges [1]. The World Health Organization (WHO) has reported that in countries in the Global North the concordance to long-term therapies stands at about 50% [2]. The administration of properly designed long-acting formulations reduces the frequency of required doses needed to achieve and maintain therapeutic efficacy, improving patient compliance and overall reducing unwanted side effects. Furthermore, depot formulations could be particularly beneficial for classes of patients that are unable to adhere to treatment regimens, such as those suffering from psychiatric disorders [3].

The remarkable number of yearly approved new parenteral molecular entities, including antibodies, proteins, and peptides, but also small molecules characterized either by instability in the gastrointestinal tract or high first-pass metabolism, encourages the design of more versatile drug delivery technologies. This review aims at providing a synopsis of the most recently developed platforms (over the last 5 years) with lipids as a primary excipient, emphasizing systems with high translational potential and offering a critical perspective on non-standardized in vitro release assays.

**Figure 1:**
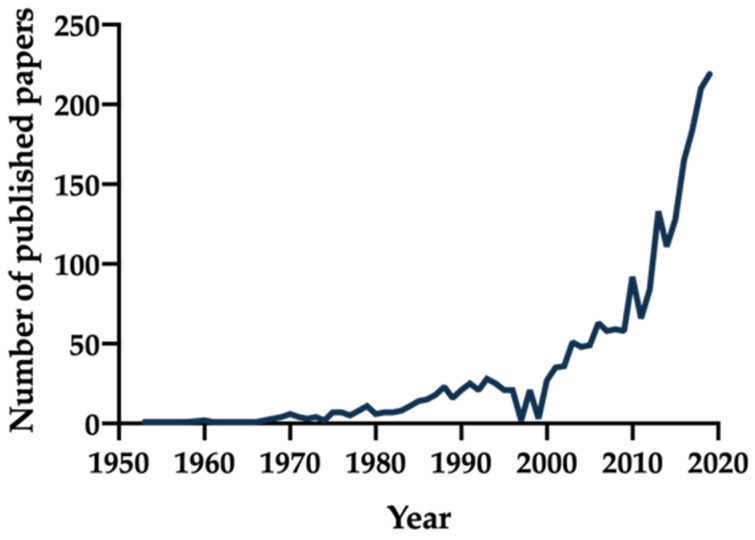
Number of publications over the years using as search terms “long acting injectable”, “long acting parenteral”, “long acting depot”, “depot formulation”, “sustained release parenteral”, or “controlled release parenteral” via a PubMed inquiry by year [6].

Despite the massive scientific output in this field, to date no consensus in the terminology has been reached. A plethora of delivery technologies for injectable depots has been developed over the last years, as shown in Figure 1, but inconsistent terms to describe the concept of long-term release are routinely employed: “long-acting injectable”, “controlled release”, “sustained release”, “extended release”, or “depot” formulations. In terms of the duration of the release, questions arise as to when a drug delivery system could be considered “sustained” or “prolonged”, or when a formulation could be denominated as a “depot”. The United States Pharmacopeia (USP) describes “extended release”, a synonym for “prolonged” and “sustained release”, as a deliberate modification to protract the release rate of an active pharmaceutical ingredient (API) in comparison to an immediate release dosage form [4]. In the European Pharmacopoeia (Ph. Eur.), prolonged release and extended release are used as synonyms and are defined as characterized by a slower release of the API with respect to a conventional release dosage form administered by the same route [5]. It is still unclear whether even a prolongation of a few hours could be considered as a sustained release or if only a protraction expressed in days is noteworthy. Although a handful of examples of depot injectables able to extend release only for few hours are mentioned in the present review, the majority of technologies we considered provide a sustained release of the API in a timespan of days at least.

## 2. The Ideal Depot Delivery System

An ideal depot formulation delivers the drug at a tunable, predetermined rate within the therapeutic range for a specified period, ideally for as long as possible for the treatment of chronic diseases [7]. Release can take place directly at the site of action for a local treatment or at a systemic level, thus reducing the adverse side effects of the drug to a minimum [8]. Furthermore, the ideal delivery system undergoes full biodegradation at a rate consistent with the desired release rate of the API, and the matrix biocompatibility does not induce adverse reactions at the site of injection [9]. During the development of a novel delivery system, an adequate shelf life has to be considered in order to enable the desired release profile and longevity. During storage, the loaded drug does not leak from the vehicle in order to avert a burst release after application and to ensure that the entire dose is released over the desired time range. Interactions of the drug with the delivery systems could be exploited to improve stability and to reduce leakage of drug, although it has to be ensured that such a stabilization strategy does not interfere with the desired release profile [10]. 

In practice, not all requirements could be fulfilled in a universal delivery platform; nevertheless, existing lipid-based systems are continuously being improved to meet as many conditions as possible, as highlighted in the following sections.

### 2.1. Excipients for Formulating Depots

Two major classes of chemical compounds are used to create drug delivery systems: polymers and lipids [11]. Despite the fact that both serve the same purpose—encapsulating drugs for depot formulations—they differ in their fundamental properties like chemical structure, solubility, or biodegradability and biocompatibility. The focus of this review is exclusively on depot formulations based on lipids, although hybrid systems combining the benefits of lipid and polymeric excipients will also be discussed. For more details about polymeric drug delivery systems we refer the readers to dedicated comprehensive recent reviews [12,13]. 

Lipids are defined as hydrophobic or amphiphilic small molecules originating entirely or in part by carbanion-based condensations of thioesters and/or by carbocation-based condensations, and are classified by LIPID MAPS^®^ into eight categories shown in Table 1 [14,15]. 

### 2.2. Parenteral Administration Routes for Depot Injectables

Administering depot formulations parenterally is a recommended choice for drugs which undergo substantial first-pass metabolism or that are characterized by low oral bioavailability. Typically, the subcutaneous (s.c.) and intramuscular (i.m.) routes are preferred to achieve a systemic effect. When local treatment is sought, intraarticular or intraocular injections are also considered viable options depending on the condition to address. The absorption, distribution, metabolism, and elimination of a drug strongly rely on the characteristics of the biological environment. Opting for a specific site of administration thus represents a strategy to modulate the drug release kinetics. As the blood perfusion in the s.c. tissue is lower than in the muscles, a s.c. injection will result in a delayed absorption of the drug with respect to an i.m. injection [17]. Targeted, localized drug delivery could be beneficial for diseases affecting only a small, distinct area of the body, resulting in a high local drug concentration but a low systemic concentration, thus reducing systemic side effects and overall toxicity. Moreover, diffusion barriers and drug metabolism could be circumvented. 

S.c. injections are the most-often used parenteral administration route due the possibility of self-injection by the patients, but the administered volume is limited. The administration of volumes larger than 1.5 to 2 mL usually requires multiple injections to minimize the risk of considerably high pain upon injection. For example, it has been shown that incrementing the injected volume from 0.8 to 2.25 mL resulted in an increase of the perceived pain about 1.7-fold [18,19]. The choice of the most suitable drug delivery system has to be carefully considered; the potency of the drug and the dose on the one hand and the encapsulation capacity of the delivery system on the other play a decisive role in this selection. Although it has been reported that the used buffers (pH, osmolarity) in the formulations could influence the pain upon injection [18], no correlation between different lipids and the induction of pain has been investigated so far.

Sterilization of the final product is mandatory for this route of application; however, (phospho)lipids are sensitive to high temperatures, and sterilization processes such as steam sterilization or dry heat sterilization may cause oxidation and hydrolysis of the (phospho)lipids, resulting in a destabilization of the delivery system. Sterilization of lipid-based products might thus be achieved via gamma-irradiation, sterile filtration (only for colloidal systems with a size below 200 nm), or via production under aseptic conditions [20].

### 2.3. Biocompatibility

Host response to the depot formulations, including foreign body reactions or fibrous encapsulation, plays a pivotal role in the choice of materials for long-acting formulations. The extent is dependent on the size, shape, and composition of the drug delivery system on the one hand and on the targeted tissue or organ on the other [21]. An initial acute inflammatory response is triggered after every injection due to the minimal injury of the connective tissue. Released leachables or degradation products from the delivery system could promote this inflammatory response and could turn the acute inflammation into chronic inflammation if the stimuli are persistent [22]. Ultimately, a collagenous fibrous capsule of typically 50–200 µm thickness can be built as reaction of the immune system to the foreign object, especially for solid implants or in situ forming systems. This creates an additional diffusion barrier influencing drug release [23,24,25].

Being able to predict biocompatibility is of paramount interest for a successful delivery system: early toxicity screenings could be performed using in vitro tests but for the assessment of the multiple tissue reactions, in vivo studies combining pharmacokinetic and pharmacodynamic effects are also required. Besides quantifying the plasma concentrations of inflammatory cytokines and the macro- and microscopical inspection of the surrounded tissue, a cage implant system could be used. This system enables the characterization of degradation products of the drug delivery system as well the inflammatory exudates in the surrounded tissue. Regrettably, the inflammatory reactions from the cage itself could represent an insidious Trojan horse [26,27,28].

Lipids are characterized by an outstanding biocompatibility and safety profile due to their occurrence throughout the living world and the biodegradability in the human body induced by lipases [29,30]. For liposomes, the lipids used for the production of multivesicular liposomes did not induce any foreign body reaction [31,32].

## 3. Oil-Based Solutions

**Figure 2:**
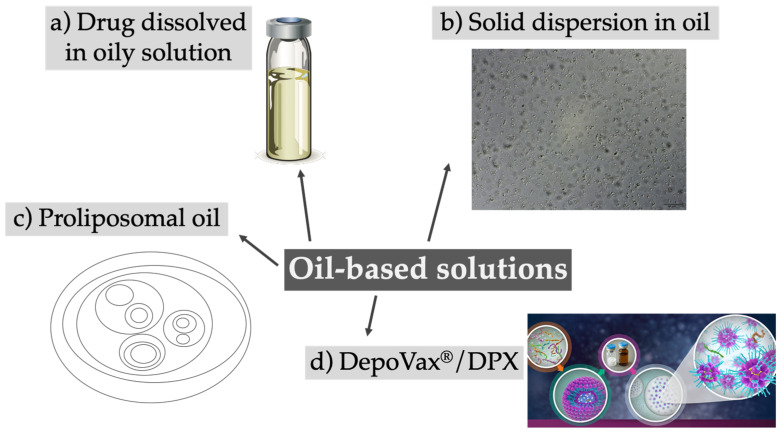
Overview of different approaches using oil-based solutions as depot formulations, ranging from the classical dissolved drugs in oil (**a**) to more advanced systems like solid dispersions (**b**), proliposomes (**c**), or the DepoVax/DPX system (**d**). (**a**) Adapted from [37]; (**b**) Reprinted from [38], with permission from Elsevier; (**d**) Adapted from [39].

Oily solutions containing a dissolved or suspended drug were first introduced in the 1950s and are hence the oldest approach proposed for injectable depots. They were traditionally used for antipsychotic drugs or hormones, and several different products became available over the decades, including Haldol Decanoate, Depo-Testosterone, Invega Sustenna, or Depo-Provera, to name a few that are still on the market [33,34]. With the aim of providing a more pronounced lipophilicity to extend drug release, highly hydrophobic esters were usually conjugated to first-generation antipsychotics. The drugs are dissolved in pharmaceutical oils like sesame seed oil or middle-chain triglycerides. Upon i.m. injection, drug transfer from the oil phase into the tissue fluid represents the main release-limiting factor. Spreading of the oil solution along the muscle fibers can occur, increasing the surface area and therefore accelerating drug release [35,36]. Although this depot system has a favorable long-term stability and can be prepared in a fast and uncomplicated manner with sterilization of the final product, only lipophilic drugs can be included. Hydrophilic drugs have to be modified prior to insertion, which limits the use of the system. In recent years almost no new approaches using dissolved drugs in oily solutions have been reported, and the system is still traditionally used for antipsychotics (Table 2). The concept of oil-based solutions has been further elaborated, and, in most cases, the drug is encapsulated in a carrier and then dissolved in an oil phase (Figure 2). 

A hybrid system based on methylcellulose microparticles suspended in corn oil was proposed by Nguyen et al. [40]. The in vitro release of encapsulated ondansetron was prolonged up to 120 h, and after s.c. injection into rats, the plasma concentration was maintained over 72 h with an enhanced bioavailability. Lu et al. tried a similar approach based on a solid dispersion of ivermectin in hydrogenated castor oil, demonstrating a prolonged release in vitro and a higher AUC (area under the curve) and mean residence time in rabbits [43,44]. Besides favorable pharmacokinetics, such an approach proved to have a therapeutic potential, as proved by the elimination of a parasite infection in rabbits treated with the ivermectin depot. An advancement of this concept was attempted for the development of a smart, stimulus-sensitive system: a micro-environmental pH-modifying solid dispersion suspended in oils for drugs with pH-dependent solubility offering sustained release of toltrazuril in vitro investigated over 72 h, with good biocompatibility [38]. This drug is only used in veterinary medicine, but the system might be applicable for other drugs used in humans. With the aim of preventing moisture contact to hydrophilic APIs during formulation, a complete anhydrous nanoprecipitation method for the hydrophilic drug disoproxil fumarate was recently reported by Hobson and coworkers [45]. The nanoprecipitates were subsequently dispersed in different oils intended for i.m. injection. In vitro release studies using a dialysis method were carried out only over 6 h, making it hardly possible to estimate the depot effect. An additional drawback of the system is represented by the use of organic solvents like dichloromethane and methanol in the manufacturing process.

The above-mentioned approach was revisited by the company PainReform Ltd. (Herzeliya, Israel): phospholipids, co-dissolved in an oily solution with a drug of choice, created a proliposomal oil, thus transforming a classical drug delivery system in a pro-formulation system [41,42]. Upon injection and contact with body fluids the amphiphilic phospholipid molecules self-assemble into drug-loaded multilamellar liposomes in the micron range. Following s.c. injection in pigs, a proliposomal oil formulated with ropivacaine showed a 5-fold increase in half-life and local anesthesia effect, while in human volunteers the formulation of ropivacaine in a proliposomal oil resulted in a 2- to 3-fold increase in anesthetic effect over plain ropivacaine. Combining the advantages of liposomes with an oil-based solution represented the core idea of a vaccine delivery platform with sustained immunological activity. The system, called DPX (formerly DepoVax™), is based on lyophilized liposomes encapsulating antigens or nucleic acids which are dispersed in oil diluents [48]. In comparison to regularly used emulsions, the system does not require the use of emulsifiers, does not support a passive release in vitro, and retains the antigens at the site of injection in vivo. DPX facilitated in vivo the development and persistence of T cells and provided a better control of tumor growth [46]. Recently, Weir and coworkers used DPX for an anthrax vaccine formulation to reduce the current multidose vaccination plan (five injections over 12 months combined with an annual booster) to a single injection, obtaining 100% protection against anthrax in rabbits and non-human primates [47]. Different clinical trials in early or late phase 2 are ongoing for the treatment of ovarian cancer and lymphoma using immunotherapy [49]. 

Although the preparation of oily solutions might be easy and fast, these depot systems have the disadvantage of spreading at the site of injection because of their low viscosity. Consequently, semisolid formulations with a higher viscosity or liquid in situ solidifying systems were developed (see Section 5, Semi-Solid Preparations and in Situ Forming systems). The latter approach especially combines the advantages of easy administration and the formation of a physical depot in vivo (vide infra).

## 4. Liposomal Systems

**Figure 3:**
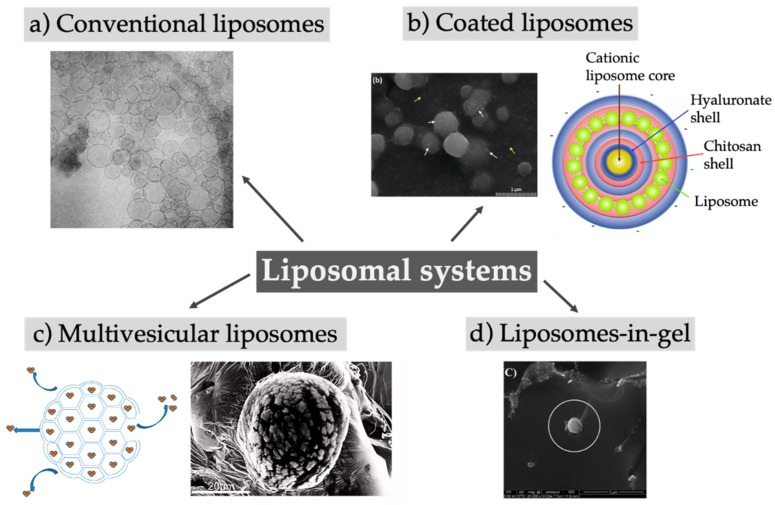
Overview of different approaches using liposomal systems like large unilamellar liposomes (**a**), multivesicular liposomes (**c**), liposomes with different coatings creating core-shell-systems (**b**), or the embedding of liposomes in gels (**d**). (**a**) Reprinted from [55]; (**b**) Reprinted from [56] and (**d**) reprinted from [57] with permission from Elsevier; (**c**) Adapted from [50,58].

First described in 1961, liposomes are spherical vesicles composed of a phospholipid bilayer enclosing an aqueous core. In addition to phospholipids, other lipids like cholesterol can be incorporated into the bilayer, while the surface of the vesicles can be modified with sterically stabilizing polymers and/or targeting ligands [50]. Liposomes are traditionally used as a drug delivery system to increase the solubility of hydrophobic drugs, reduce their toxicity, prolong the circulation in the blood, or enable active targeting [51]. Alone or in combination with other excipients (hybrid systems), liposomes may provide a sustained release of the encapsulated drugs [52,53,54]. The versatile applicability of the system is depicted in Figure 3 and described in Table 3.

The release of drugs encapsulated in liposomes, and with it the possibility to achieve a depot effect, is mainly dependent on the diffusion of the drugs across the bilayer. By generating liposomal aggregates, the diffusion of the drug into the surrounding can be mitigated, as recently proposed by our group [55]. Long-acting drug delivery systems could indeed be generated in situ, controlling the aggregation of liposomes formulated with negatively charged phospholipids (NCPs) through the interaction with divalent cations. Various NCPs were screened as function of their head groups and acyl chains for their suitability as scaffold for depot formulations and the system extensively characterized from a physico-chemical viewpoint. Mixing liposomes formulated with either 1,2-dipalmitoyl-sn-glycero-3-phosphate (DPPA) or 1,2-distearoyl-sn-glycero-3-phospho-(1′-rac-glycerol) (DSPG) with physiologically compatible concentrations of calcium and magnesium resulted in a rapid fusion-free aggregation, confirming the pharmaceutical potential of intact liposomes as single composing units of the depot. Bupivacaine-loaded liposomal aggregates are presently under evaluation in our laboratories as an analgesic platform alternative to the current strategies for pain management.

Negatively charged phospholipids could be also used to form so-called cochleates, defined as supramolecular assemblies of phospholipid bilayers which form spiral structures with divalent cations [82]. Despite the thorough characterization conducted by Fahr and coworkers so far [83,84], their clinical potential regarding their use as drug delivery system still needs to be investigated. Drawing from this concept, Blazaki et al. induced aggregation of negatively charged liposomes with protamine and encapsulated two different fluorescence dyes or the anti-inflammatory drug flurbiprofen [59]. The in vitro release of the fluorescent dyes was sustained for more than 17 days, whereas flurbiprofen showed a sustained release over only 120 h. Despite the increase in retention of flurbiprofen in vivo in the posterior eye segment after intravitreal injection achieved with the aggregated liposomes, the differences with respect to the non-aggregated system were rather small. Seeking a strategy aimed at avoiding repeated injections during emergency treatments such as cardiopulmonary resuscitation, Schlich et al. described the use of adrenaline-loaded PEGylated liposomes [60]. One of the reported challenges during the production process of this formulation was to prevent the oxidation of adrenaline, which could ultimately be ensured by addition of an antioxidant while maintaining the loading efficiency of the active loading method. The in vitro release of the API was investigated for only 8 h (cumulative release of around 10%) in two different release setups, with neither reflecting the complexity after intravenous injection. The authors did not address how this liposomal system could be included in the treatment protocol for emergency situations in clinical practice.

Liposomes could not only act as a sustained drug delivery system, but the phospholipids themselves could exert local, mechanical beneficial effects in the management of osteoarthritis [62]. Distearoyl phosphatidylcholine (DSPC) liposomes encapsulating D-glucosamine sulphate combined the anti-inflammatory effects of the drug and the lubrication ability of the liposomal system in the joint. A prolonged release over several days in vitro was achieved and the friction between a silica and polystyrene surface in an atomic force microscopy setup reduced. The lubrication effect of the liposomes is attributed to a hydration layer around the phospholipid headgroups creating a ball-bearing-like system. It is questionable whether the lubrication enhancing is a clinically relevant effect, since, so far, this has only been tested in instrumental setups or ex vivo using human cartilage [85,86].

A combination of fast and prolonged releasing liposomes was used by Christensen et al. as a parenteral vaccine strategy able to generate a long-lasting intestinal immune response to prevent enteric infections [61]. The fast releasing system comprised retinoic acid in PEGylated liposomes to pre-condition the vaccine-draining lymph nodes. Cationic liposomes based on the surfactant dimethyldioctadecylammonium bromide (DDA) and stabilized with the synthetic immunostimulator trehalose 6,6′-dibehenate (TDB) formed a depot at the site of injection and delivered over a prolonged period of time the antigen. In vivo results confirmed this hypothesis and the combination of both species induced an antigen-specific intestinal immunoglobulin A response after s.c. injection.

Looking at the list of the liposomal drugs approved by the US Food and Drug Administration (FDA), only three can be strictly considered sustained drug delivery systems: Exparel^®^ (bupivacaine), DepoDur^®^ (morphine), and DepoCyte^®^ (cytarabine). These three formulations share an important aspect, that is, the same multivesicular liposomes technology, DepoFoam^®^ [87,88]. Multivesicular liposomes (MVLs), first described by Kim et al. 1983, are micron-sized vesicles containing numerous internal aqueous compartments divided by non-concentric lipid bilayers, as shown in Figure 3c [89,90]. The production follows a two-step, water-in-oil-in-water double-emulsification process under aseptic conditions [91]. In contrast to conventional liposomes, the use of a neutral lipid besides phospholipids is mandatory to form this unique structure [89]. Several mechanisms that contribute to the release of encapsulated drugs, like diffusion, erosion, and reorganization of the compartments as well the used phospholipids and neutral lipids have a major influence on the release profile [89,92]. While DepoDur and DepoCyt were discontinued from the market in the United States or Europe, Exparel was recently approved (2011) [87,88,93]. The research approaches aiming at exploring this technology for the delivery of bupivacaine in new indications are proliferating [71,72,94]. Besides bupivacaine, ropivacaine was considered as cargo for MVLs for prolonged-duration local anesthesia [65,66]. After successfully encapsulating small molecules, attention was drawn to the use of this delivery system for biomacromolecules. MVLs loaded with bevacizumab were used for the treatment of choroidal neovascularization, resulting in a prolonged intravitreal retention time of bevacizumab and consequently in a reduced number of intraocular injections [58]. Becavizumab–MVLs turned out to be superior to plain bevacizumab solution with respect to the effective inhibition of the thickness of the choroidal neovascularization lesion at 28 days after treatment. The MVLs showed a prolonged release for 10 up to 13 days in vitro, mostly sustained for the formulation containing 1,2-dioleoyl-sn-glycero-3-phosphocholine (DOPC). Interestingly, a more extended depot effect using conventional liposomes was reported by Karumanchi et al. [63]. Bevacizumab was released in vitro over 70 to 100 days out of conventional liposomes and over an even much longer time period using stealth liposomes (150 to 200 days). Besides differences in the in vitro drug release setup, the in vivo experiments were designed differently, and thus the results could not be compared directly. Karumanchi et al. injected the conventional liposomes into the anterior segment of rabbit eyes and observed therapeutic concentrations of bevacizumab over 22 weeks, as determined by non-invasive fluorescence imaging after fluorescence tagging of the antibody. Bevacizumab–MVLs injected into rabbit eyes showed therapeutic concentrations over the full 56 days of study duration after direct measurement of the antibody concentration in vitreous and aqueous humor. The comparison of these two studies underlines how critical the choice of the in vitro release setup is to compare results obtained from different groups.

Although mostly hydrophilic drugs were encapsulated very efficiently owing to the large aqueous compartments in the MVLs, various attempts were made to include hydrophobic drugs into this system. Luo et al. encapsulated oleanolic acid, a pentacyclic triterpenoid, into the bilayer of the MVLs and in vitro and in vivo showed a prolonged release and plasma concentrations superior to free oleanolic acid, respectively [68,69]. A different approach to include a hydrophobic agent into the MVL was pursued by Vafaei and coworkers using cyclodextrins [70]. The lipophilic model drug fluocinolone acetonide was encapsulated into different cyclodextrin inclusion complexes and subsequently incorporated into MVLs by reverse-phase evaporation. The in vitro drug release was sustained up to 180 h depending on the used cyclodextrin.

To summarize, MVLs are an all-round platform for prolonged drug delivery. A high encapsulation efficiency for hydrophilic drugs can be achieved due to their relatively high water content (aqueous:lipid ratio 95:5). The nonconcentric bilayer geometry increases the stability of the system and reduces a burst release, while the depot effect itself relies on reduced clearance, an aftermath of the micron-range size of the vesicles [87,91]. Despite the superb clinical success, the drawbacks of this system, mainly with respect to the manufacturing procedure, should not be overlooked. The double-emulsification process requires the use of organic solvents and needs to be performed entirely under aseptic conditions [89,90].

At the other end of the scale, conventional small and large unilamellar liposomes are still being intensively investigated and show tremendous potential for depot systems, especially in a hybrid modality, in combination with other biocompatible materials like gel matrices to form liposomes in gel, which are favorable for local drug release due to the increased viscosity of the system. The biodegradable hydrogel poly(lactic-co-glycolic acid)–polyethylene glycol–poly(lactic-co-glycolic acid) (PLGA–PEG–PLGA) was proposed as matrix to embed liposomal doxorubicin, thus enabling the taming of the burst release typical of PLGA systems in favor of a smoother, prolonged release [76]. For the first 12 h the hybrid composite depot outperformed the pristine doxorubicin in hydrogel, while the in vitro sustained profile of the liposomal gel was maintained for over 11 days; furthermore, tumor growth was significantly reduced in a breast cancer model in mice, and the known cardiotoxicity of doxorubicin was reduced. The same approach of liposomes in gel but using a Pluronic^®^ (poloxamer) hydrogel was tried by Fu and colleagues, who encapsulated doxorubicin and paclitaxel for the treatment of hepatocellular carcinoma [73]. After local injection the system inhibited the tumor growth in vivo significantly and reduced the toxicity of the drugs.

Coating the liposomal surface with polymers could represent an alternative strategy to extend drug release over time, as proposed by Ravar and coworkers [80]. Hyaluronic acid was used produce hybrid polymer-lipid vesicles via electrostatic coating upon titration of 1,2- dioleoyl-3-trimethylammonium-propan (DOTAP)-based large unilamellar liposomes encapsulating paclitaxel. Although the in vitro release of paclitaxel followed over 40 h was not substantially prolonged in comparison to hyaluronic acid-free plain liposomes, the tumor growth in vivo in 4T1-tumor bearing mice intravenously injected with the depot system was reduced by about 10-fold with respect to the ones treated with a commercial paclitaxel solution, and the overall survival of the mice increased by 60% over 20 days. The addition of more than one polymer can lead to the formation of layer-by-layer particles, also called capsosomes, arising from electrostatic interactions [56]. The capsosomes were formed by covering a cationic liposomal core layer-by-layer with hyaluronic acid and chitosan, topped with small sized liposomes and hyaluronic acid. Although it seems a promising approach to sustain the drug release using several layers as a diffusion barrier, investigations about the encapsulation of drugs and their release are missing. A completely different strategy was adopted by Nascimento Vieira et al., who encapsulated a drug into cycodextrins and included this complex into liposomes to improve the encapsulation efficacy, prolong the release, and enhance the biological activity [81]. The ternary drug delivery system containing ropivacaine sustained the release in vitro up to 8 h while the cell toxicity of ropivacaine was reduced. Furthermore, the in vivo sensory block of the local anesthetic was prolonged 1.7 times in comparison to the drug solution. These examples demonstrate the variety of liposomal hybrid systems acting as an alternative to MVLs, and their clinical relevance should be further investigated.

## 5. Semi-Solid Preparations and In Situ Forming Systems

**Figure 4:**
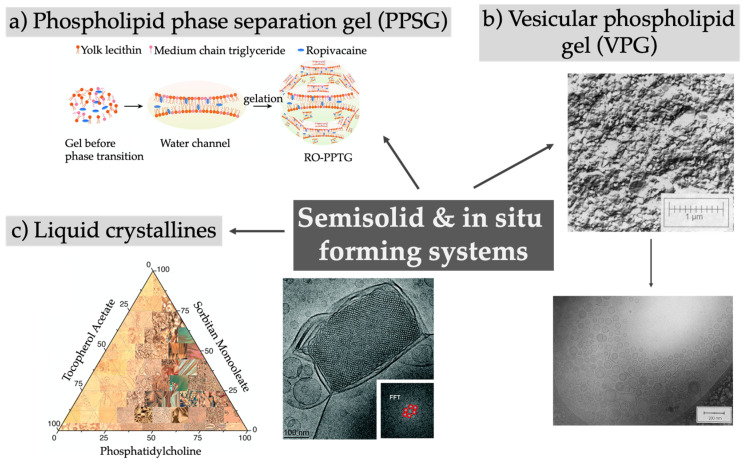
Overview of semisolid and in situ forming depot systems, for example phospholipid phase separation gels, vesicular phospholipid gels, or liquid crystalline phases. (**a**) Reprinted from [95]; (**b**) top image reprinted from [96]; (**b**) Bottom image reprinted from reprinted from [97]; (**c**) Left image reprinted from [98] with permission from Elsevier; (**c**) Right image published by The Royal Society of Chemistry [99].

Injectable in situ forming gels are an attractive way to deliver drugs due to their easy handling prior to and during administration. The depot can be conveniently generated upon contact with body fluids or when the physiological temperature is reached upon injection. A brief overview of the reported technologies is given in Figure 4 and summarized in Table 4.

Phospholipid-based phase separation gels (PPSG) combine simple manufacturing with the use of excipients known for their high biocompatibility and of solvents with low toxicity such as ethanol, soybean oil, and medium-chain triglycerides. By dissolving the phospholipids in ethanol and neutral oil mixtures and subsequently adding the drug of choice, a straightforward upscale can be envisaged, while a sterilization by sterile filtration can be performed to ensure the production of a product suitable for parenteral administration [100,104]. After s.c. injection and contact with the body fluids, a sol–gel transformation occurs due to the diffusion and exchange of ethanol out and tissue fluids into the gel, initiating a phase transition of the phospholipids and resulting in an increase in the viscosity in a range from 10- to 10^6^-fold [95,103,104,110]. The remarkably higher phospholipid concentrations (up to 70%) of PPSGs with respect to organogels or vesicular phospholipid gels is responsible for the low viscosity and negligible initial burst release after administration [104]. The existence of the gel over a long time period was proven by fluorescence imaging or harvesting of the depot [95,100,103,105]. Encapsulating small molecules could prolong the release in vitro for several days, as shown by Li et al. when incorporating ropivacaine into PPSG [95]. The initial burst observed for the local anesthetic solution after s.c. injection into rats was reduced using the depot system and the nerve blockade in guinea pigs lasted three times longer. The in vivo drug release or the therapeutic effect for biomacromolecules was observed for up to weeks, as in the case of exenatide released over 3 weeks [106], and leuprolide acetate and octreotide acetate both released for over 4 weeks [107,108]. The administration of an antigen using this delivery system led to a persistent immune response and immunologic memory, showing the potential of PPSG as new vaccine adjuvant [105]. In general, foreign body reactions and tissue responses are related to the ethanol content and are reduced by increasing the phospholipid content, while the inflammatory responses are low and disappear with depot degradation [100,103,105]. Using this depot system, the toxicity and side effects of the drug itself are reduced while showing the same or even better therapeutic effect [101,102,105]. PPSGs are fully biodegradable but only limited information is available about the detailed degradation pathway. The formation of small vesicles at the edges and their release from the main gel are assumed [100].

In comparison to the PPSG, vesicular phospholipid gels (VPGs) comprise phospholipids not in solution, but rather as tightly packed vesicles and lamellar structures. This minimizes the surrounding aqueous phase and leads to the typical semisolid behavior mediated by the steric interactions of the vesicles without the need for additional viscosity enhancers. Dilution of the system after injection results in the formation of small unilamellar vesicles, as recently reviewed [136]. The inner structure of the densely packed vesicles causes a relatively high viscosity and it might be difficult or even impossible to inject them. A promising needle-free injection approach for VPGs to overcome this hurdle was proposed by Breitsamer et al., but a clinical validation is to date still missing [137]. VPGS have been intensively investigated for the delivery of proteins and peptides [111,112,113]; for example, exenatide–VPGs showed a release in vivo over 11 days, and the hypoglycemic effect in diabetic rats lasted for 10 days upon injection [114].

Latitude Pharmaceuticals Inc. offers a phospholipid gel depot (PG Depot) that contains between 20% and 80% phospholipids nano-dispersed in water and can be easily injected through a 25 G needle. A variety of drugs can be loaded in this system, from small molecules to proteins and peptides. In a first step a phospholipid nanodispersion with a high-water content is produced and a gel phase is subsequently obtained by removing water in a second step. The depot effect could be adjusted from 1 to 7 days [116,117,138].

Lyotropic liquid phases are characterized by a self-assembly of amphiphilic lipids to different lamellar, hexagonal, or cubic phases or a reversed micellar cubic phase. The relatively high viscosity of the systems is an advantage for topical administrations but is not beneficial for their injectability. Consequently, the cubic or hexagonal phase can be dispersed in aqueous solutions forming cubic (“cubosomes”) or hexagonal nanoparticles (“hexosomes”) with an intact inner liquid crystalline structure and larger surface area but a lower viscosity [139]. An alternative strategy to improve their injectability is based on the use of a lower amount of viscous precursor (with a low amount of water): body fluids are taken up in vivo upon injection, resulting in the formation of a viscous depot system in situ [118,123,125]. A comprehensive overview about the detailed inner structure, preparation methods, and applications has been given in two recent reviews [140,141]. Narrow-sized water channels in the liquid crystal structure cause the sustained release behavior of low-molecular-weight drugs but might inhibit the release of molecules with higher molecular weight and therefore demand the addition of hydration-modulating agents [142]. The in vitro duration of action of the depot effect ranges from several days for bufalin [125], to 10 days for minocycline [126], or up to one month for leuprolide acetate [98], and can be easily tuned by adjusting the ratios of the used compounds [121]. A novel non-viral gene delivery system was designed by Borgheti-Cardoso et al., combining liquid crystals with polyethylenimine (PEI) to complex siRNA (small interfering RNA) [129]. The release of siRNA was maintained over 7 days in vitro, while in vivo the in situ formed gels were degraded over 30 days, inducing only a mild inflammatory response. The siRNA was released in a complex with PEI, which protects the siRNA against degradation, but experimental data about the gene silencing efficiencies of the released siRNA are missing to date. Overall, the groups developing liquid crystalline phases as injectable depots have focused mainly on the characterization of the systems using methods like small angle X-ray scattering, electron or polarized light microscopy, rheology, and in vitro drug release studies [98,124,143]. Besides measuring drug plasma concentrations, little attention has been paid to additional in vivo characterization like the fate of the depot after injection, possible local side effects, and biocompatibility or the pharmacological effect.

The only liquid crystalline system that has reached clinical studies is based on the FluidCrystal^®^ technology marketed by Camurus AB. Recently, Buvidal^®^ (buprenorphine weekly or monthly) was approved in the European Union and Australia and tentatively approved in the United States (Brixadi™) for the treatment of opioid use disorder [130,131]. The depot system is based on a phosphatidylcholine and glyceroldioleate precursor solution which forms in situ a reversed non-lamellar liquid crystalline structure with a tunable release profile [144,145]. Other products in different phases of clinical studies, such as octreotide for the treatment of acromegaly and neuroendocrine tumors [119,132], leuprolide for the treatment of prostate cancer, or setmelanotide for treating genetic obesity [120], demonstrate the great potential of liquid crystalline phases as depot system.

Although the aqueous-based nanoemulsion developed by Rachmawati et al. does not show a semi-solid behavior, it is discussed in this chapter since it relies on the same principle of dispersed lipids in aqueous solutions [133]. Bupivacaine is distributed in castor oil droplets stabilized by polymeric surfactants, as proven by cryo-transmission electron microscopy. Different biodegradable oils and polymeric surfactants were evaluated for their potential as depot formulations but only the nanoemulsion prepared with castor oil showed sufficient stability over 3 months. The release of bupivacaine was extended in vitro up to 24 h. Evaluating the in vivo plasma profile, the initial burst release using the nanoemulsion was lowered in comparison to the bupivacaine solution, but the nanoemulsion containing the same amount of bupivacaine as the control solution showed a lower analgesic effect than the bupivacaine solution (as explained by the slow release), and did not achieve an effective concentration. After increasing the concentration of drug in the nanoemulsion three times, the analgesic effect was then prolonged up to 24 h.

Classical organogels merely based on immobilized organic phase in a three-dimensional network-building gelling agent undergo sol–gel transition in situ due to temperature increase or because of partial desolvation [146]. Although in recent years major scientific efforts have been made to expand the current portfolio of in situ forming systems, considered very attractive for their superior clinical translational properties, some attempts to synthesize novel organogelators were successfully carried out. For example, Li and coworkers synthesized a novel low-molecular-weight bis-amide organogelator able to prolong the release of candesartan cilexetil out of a soybean organogel for up to 10 days in vivo with reasonably moderate inflammation at the injection site [134]. Newly synthesized organogelators based on amino acid derivates induced a prolonged release in vitro of risperidone out of soybean oil [135]. The depot effect was confirmed in vivo, showing an almost linear release behavior after s.c. injection over 8 days and suggesting a possible relevant translational potential, but a comprehensive toxicity and biocompatibility study is missing.

## 6. Solid Particles and Implants

Solid drug delivery systems could be produced according to different technologies, each resulting in products varying in size and structure.

**Figure 5:**
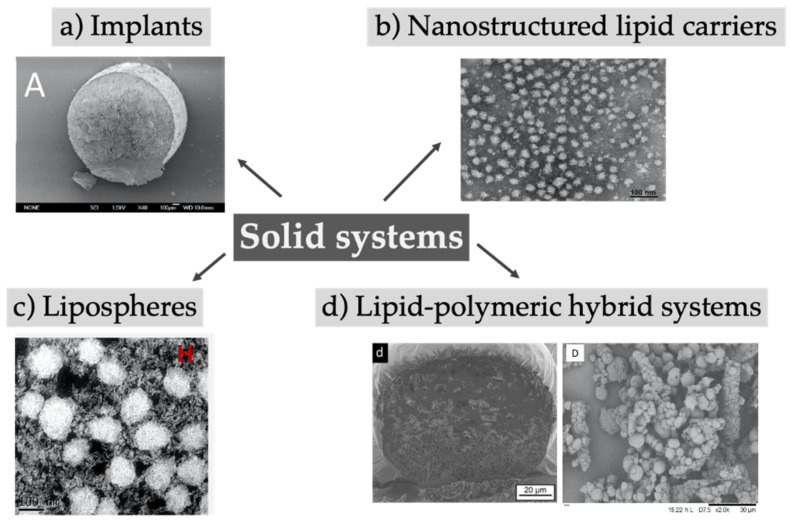
Depot systems based on lipid implants or solid particles made of lipids or mixture of lipids and polymers. (**a**) Reprinted from [151]; (**b**) Reprinted from [152]; (**c**) Reprinted from [153]; (**d**) Right image reprinted from [154] with permission from Elsevier; (**d**) Left image adapted from [155].

Solid lipid implants are monolithic, typically cylindrical-shaped systems with diameter and length in the millimeter range, whereas solid lipid nano- or microparticles are characterized by a size in the nano- or micron-range, respectively, and both systems contain lipids or mixtures of them in a solid state (Figure 5). To overcome the physical instabilities and the short shelf-life often associated with solid lipid nanoparticles, nanostructured lipid carriers were introduced and are characterized by a less ordered crystal structure due to the addition of liquid lipids to the solid lipids [147,148]. Surrounding classical lipid cores with a phospholipid bilayer, lipospheres were created which were designed to reduce the production costs of liposomes and improve their stability. The absence of an aqueous core compared to liposomes limits the encapsulation of drugs to only lipophilic substances [149]. Despite the fact that most often preparation requires the use of organic solvents, a method averting the use of toxic organic solvents has been recently reported [150]. Furthermore, the solid lipid particles could be merged with polymers to improve stability and encapsulation efficiency, and prolong the release rate. An overview of reviewed solid lipid systems investigated in the last years is given in Table 5.

Commonly marketed biodegradable implants based on the use of polymers such as PLGA produce acidic degradation products which affect the stability of proteins and peptides [168,169]. This drawback could be overcome by using lipids for the formation of implants: no pH change during storage takes place and no burst release of the encapsulate drug is envisaged, thus providing enhanced stability of proteins and peptides. Manufacturing of lipid implants by compression was the production method of choice in the early stages of this technology because it was considered fast and uncomplicated [159,170]. Production is nowadays most commonly based on holt melt extrusion (also named solid lipid extrusion), either in single- or twin-screw devices, a process that enables the achievement of a more sustained release and homogenous drug distribution in comparison to other methods. Often-encapsulated biologics are temperature-sensitive, so the lipid mixture must be carefully selected to ensure extrusion at lowest possible temperatures [151,156,157,158,160]. Production by melting and casting lipid blends with drugs is no longer state of the art since it employs higher temperatures than those used in extrusion processes and could therefore cause instabilities in the APIs even faster [171,172]. General insights into the effect of lipid composition and different preparation parameters on the physico-chemical characteristics and the in vitro release behavior (up to 28 weeks) were obtained using albumin or monoclonal antibodies as model proteins [156,157,158,160]. Lipid implants including a monoclonal antibody from the IgG_1_ class and the f_ab_-fragment ranibizumab showed a sustained release in vitro of around 120 days with a good stability of the proteins, which were stabilized by cyclodextrines [151]. Overall, recent studies mainly focus on the preparation and characterization of the implants and do not focus on the implementation in vivo and the drug release, pharmacokinetics, or pharmacodynamics. An exception is represented by Even and coworkers, who showed in vivo results using the tumor peptide TRP2 as an antigen in lipid implants [158]. The mice receiving this implant had a delayed tumor growth of three days compared to control groups without TRP2 treatment. They explained this low effect with the slow release out of the implant and reported that this type of therapy might be not appropriate for fast-growing tumors.

Solid lipid nano-/microparticles or nanostructured lipid carriers are not commonly used for parenteral depot injectables owing to a relatively fast release caused by erosion of the particles, a phenomenon that makes them ideal candidates predominantly for topical or oral administration of drugs [161]. Nevertheless, they have been investigated as parenteral depot formulations. Nanostructured lipid carriers encapsulating ondansetron were shown to enable an in vitro sustained release up to 96 h, a result confirmed in vivo without the appearance of a burst release [161].

To prolong the release rate, hybrid systems of polymers and lipids were explored. Merging poly-ε-caprolactone nanoparticle cores with encapsulated ropivacaine and a lipid shell prolonged the release of ropivacaine in vitro to 96 h [162]. The median duration of analgesia in vivo was maintained over 36 h for the nanoparticles compared to 0.5 h for the plain ropivacaine solution. The same idea was proposed by Ma and coworkers encapsulating bupivacaine into PLGA nanoparticles which were surrounded by a lipid shell [165]. The hybrid system prolonged the in vitro release up to 96 h and, in comparison to pure polymeric nanoparticles, the initial burst release was reduced. This observation was confirmed in vivo with a 5-h extended analgesic effect of the polymer–lipid hybrids over the polymeric nanoparticles. The influence of different lipid types on core–shell particle systems was demonstrated using mannitol microparticle cores with embedded monoclonal antibody IgG_1_ [163]. Glyceryl stearate as a coating showed an incomplete in vitro release of IgG_1_, whereas the release profile was prolonged up to 6 weeks with increasing amounts of hard fat as the lipid shell and the initial burst decreased.

Besides being used to create core–shell systems, lipids could also be included into the matrix of polymeric systems with the aim of modulating the release profile of co-loaded drugs. For example, encapsulating risperidone in PLGA microcapsules formulated with tuned middle-chain triglycerides resulted in an almost ideal zero-order in vitro release kinetic over 60 days without the lag phase typically observed for pure polymeric microparticles [155]. Imaging techniques suggest a multicore inner structure of the microcapsules, with a core of dispersed lipid phase in which the drug could be dissolved. To date, in vivo results to prove the concept are still lacking. The type of triglycerides used to form hybrid particles has a major influence on the release behavior, as demonstrated by Wu and coworkers [154]. The length of the acyl chain and the amount of lipid employed were correlated with the initial burst release of lysozyme as the model protein; the longer the chain length the greater the initial burst, and, as shown for one lipid, the burst release was increased by increasing the lipid proportion from 33% to 67%. The amount of lipid influenced the encapsulation efficiency as well; depending on the type of lipid the best results were achieved for lipid proportions between 5% and 20%, without a clear trend.

Analogous to the liposome-in gel-approach, lipid nanoparticles can be embedded in hydrogel matrices to prolong the release of the encapsulated APIs. With the aim of creating a bio-shielding in situ forming gel, quetiapine-loaded lipospheres were incorporated into a thermoresponsive gel composed of Poloxamer^®^ 407 [166]. Almost similar release profiles with a sustained release over three days were achieved either with the lipospheres themselves or with the thermoresponsive gel containing lipospheres. However, in vivo studies presented almost no differences in the AUC between the lipospheres and the drug solution, but the presence of the thermoresponsive gel was crucial, protecting the lipospheres against degradation by lipases and increasing the AUC threefold, demonstrating the beneficial effects of the combination of gel and lipospheres. Embedding nanostructured lipid carriers containing estradiol valerate into a thermo-reversible hydrogel prolonged the release in vitro, with only 20% of the drug released after 56 h [167]. The in vivo evaluation revealed a rapid initial release, although the bioavailability was increased 17-fold and the t_max_ was delayed for 2 h in comparison to a commercial drug suspension, showing the necessity for improvement to manipulate the release profile.

In summary, approaches proposed for solid long-acting formulations are very diverse and range from nanoparticles to implants in the macro scale, demonstrating once again the versatility of lipid-based technologies.

## 7. From Design to Application: Considerations about In Vitro Release Testing

In most of the new depot formulations discussed above, the in vitro release behavior of the drug was tested as a first step to predict the in vivo performance and estimate the potential for clinical applications. Medicine agencies worldwide have not issued any specific requirements for testing the release from parenteral depot formulations yet, with only the standard dissolution apparatus from the USP or the Ph. Eur. being mentioned. A team from the FDA recently addressed the variations in the release behavior among the standard methods, and even for a marketed formulation like Exparel a consensus on standard in vitro release test is missing [173]. Completely different release profiles for bupivacaine-loaded multivesicular liposomes were obtained using a sample and separation method or a reverse dialysis method, and temperature and agitation speed modulated the release profile. Presumably similar results would be obtained by testing more drug delivery systems, demonstrating that the choice of in vitro release setup as well the used media have a great influence on the results and should be carefully chosen.

To date it is unclear if a release study in a bulk fluid dissolution set up under sink conditions could reflect and predict in vivo behavior, since the injection sites in s.c. or i.m. administrations have different hydrodynamics, generally low volumes of body fluids, and various possibilities for interaction with the depot delivery system. Choosing an appropriate in vitro release method mimicking the physiological conditions of the injection site could improve the correlation to the in vivo release. We believe that there is a lack in transferring standard release methods specified by USP or Ph. Eur. to ones which are closer to physiological conditions.

For mimicking the extracellular matrix (a major component of the subcutaneous tissue), employing hydrogels appears to be the most straightforward and simple approach to simulating in vivo conditions. Agarose is usually used due to its similar porosity and water content to soft tissue [174,175], as well gelatin [137,176]. This approach could be combined with UV–VIS imaging techniques or laser-induced breakdown spectroscopy to detect the real-time release of drugs and to give provide an exhaustive insight in the release behavior [159,177,178]. The company Pion claims to have developed a device simulating the stress conditions and environmental transitions that affect a drug after s.c. injection, proposing thus a more complex approach with respect to the basic use of a hydrogel matrix [179,180]. The Subcutaneous Injection Site Simulator (Scissor) is composed of a modified dialysis chamber filled with hyaluronic acid to mimic the extracellular matrix, which is immersed in a carbonate buffer. Several parameters are tracked during the in vitro release to detect interactions with the matrix. Although this system is intended for biopharmaceuticals it could be also useful for other long-acting injectables and depot formulations. More complex in vitro release setups including adipose tissue could be achieved by using skin for release tests. Especially for lipid-based depot formulations, possible interactions with the fat tissue are conceivable. The company Genoskin developed a method to keep human skin biopsies alive for seven days [181]. HypoSkin^®^ contains all three layers of the skin and is intended for s.c. injections, aiming to get a better understanding of the release mechanism, degradation rate, and in vivo behavior.

Although some of the developed methods have been described only for polymeric systems, it is envisaged that these release assays could be adapted to any kind of depot system. The use of physiological setups for in vitro release studies could give a better prediction of in vivo behavior, and may open up possible optimizations of drug delivery systems before proceeding into in vivo studies, thus reducing, replacing, and refining the animal studies according to the 3R concept of Russell and Burch [182]. Ideally, not only the physiological conditions at the injection site should be mimicked, but also possible side effects, like an inflammatory response leading to an increase in the blood circulation and a change in the pH value. In the long-term, the building of a fibrotic capsule should be taken into account.

## 8. Conclusions

Depot formulations are an attractive way to administer drugs, with a reduced dosing frequency and a simultaneous improvement of therapeutic efficacy and compliance of patients. Starting with the oldest approach of oily solution for i.m. injection in the 1950s, the continuous and successful development of novel concepts has demonstrated that lipid-based long-acting injectables represent a versatile technology. The whole range of drugs, from small molecules to biomacromolecules like proteins, peptides, and antibodies can be encapsulated thanks to the unique properties of lipids. The increasing number of approved biologics has called for the development of new parenteral drug delivery systems. In particular, in situ forming systems like liquid crystalline depots or phospholipid phase separation gels, liposomes, and implants are used for this purpose. Overall, the two most successful approaches for lipid-based depot formulations are multivesicular liposomes (DepoFoam technology) and liquid crystalline phases (FluidCrystal technology). Products such as Exparel and Buvidal, respectively, are remarkable commercial successes stemming from those depot systems. The two platforms have generated an enthusiastic consensus in the drug delivery community, and an increasing number of research groups are attempting to bring the two concepts a step further, extending the recommended applications or loading novel APIs. A plethora of other long-acting systems have undergone constant further development in recent years, proving their suitability in several in vivo studies, and these will likely be tested in clinical studies in the future. Thanks to new and improved analytical methods it is nowadays possible to visualize the release of drugs and the degradation of the delivery system in vitro and in vivo, enabling the collection of useful information about the systems for further development. Using optimized in vitro release setups mimicking physiological conditions, a better prediction about the in vivo behavior could be obtained.

A universal depot system able to encapsulate any drug, with a tunable release, no toxicity, an easy and inexpensive manufacturing process, and long-term stability remains a Holy Grail in drug delivery. Lipids stand out from other excipients formulating long-acting systems due to their unbeatable biocompatibility and safety profile, especially phospholipids which lack toxicity thanks to their physiological presence. Besides representing an ideal building block for optimal sustained-release platforms of APIs, lipids succeed in enhancing the solubility of poorly water-soluble drugs, in increasing their stability, and in protecting them from degradation, improving their bioavailability overall. From the multitude of lipid-based technologies nowadays available, the most appropriate delivery system for the API to be delivered to treat a specific medical condition can always be identified. The versatility of the lipid excipients can duly ensure further customization, ultimately resulting in a safe injectable depot with an optimal long-acting drug release over a duration of days to weeks.

## Figures and Tables

**Table 1:** Classification of lipids according LIPID MAPS with examples of subgroups [15,16].

Category	Subgroup Examples
Fatty acyls	Fatty esters, fatty amides, fatty acyl glycosides
Glycerolipids	Mono-, di-, triradylglycerols
Glycerophospholipids	Glycerophosphocholines, -serines, -glycerols, -inositols
Sphingolipids	Sphingoid bases, ceramides, phosphosphingolipids
Sterol Lipids	Sterols, steroids, secosteroids, bile acids, and derivatives
Prenol Lipids	Isoprenoids, quinones and hydroquinones, polyprenols
Saccharolipids	Acylaminosugars, acylaminosugar glycans, acyltrehaloses
Polyketides	Flavonoids, macrolides, linear or aromatic polyketides

**Table 2:** Overview of oil-based drug delivery systems.

Principle	Encapsulated Drug	Release Time Range	Advantages (+) and Disadvantages (–)	Ref.
**Classical oil-based solutions with dissolved drug**				
Oily solution	Paliperidone palmitate, medroxy-progesterone, haloperidol decanoate, testosterone (in general antipsychotics and hormones	Up to several months, in vivo	+ Fast and easy preparation + Long-term stability + Low viscosity, easy injection – Only lipophilic drugs, otherwise modification – Spreading at injection site	[33,34]
**Drug combined with second carrier in oil-based solutions**				
Microparticles in oily solution	Ondansetron	Hours, in vitro and in vivo	+ Hydrophilic and hydrophobic drugs, biomacromolecules + Low viscosity, easy injection – Further preparation steps required – System including drug must be soluble or dispersible in oil – Spreading at injection site	[40]
Proliposomal oil	Ropivacaine	Days, in vivo		[41,42]
Solid dispersion in oil	Ivermectin, toltrazuril (veterinary)	Days, in vivo		[38,43,44]
Nanoprecipitates in oil	Tenofovir disoproxil fumarate	Hours, in vitro		[45]
Lyophilized liposomes dispersed in oil (DPX/DepoVax™)	Vaccines, antigens	Weeks, in vivo		[46,47]

**Table 3:** Overview of sustained release systems based on liposomes.

Principle (Used Phospholipids *)	Encapsulated Drug/Marker	Release Time Range	Advantages (+) and Disadvantages (–)	Ref.
**Conventional liposomes**				
Aggregation of negatively charged liposomes (DPPC, EPC, HPC, DSPG, DPPG, DOPA, DPPA)	Flurbiprofen, Calcein, FITC-dextran-4000	Days, in vitro and in vivo	+ Hydrophilic and lipophilic drugs, biomacromolecules + Reduce toxicity, targeting possible besides drug delivery + Large-scale production obtainable with simple sterilization methods – Limited amount of encapsulated drug – Small size could lead to rapid releas – Fast clearance or lymphatic uptake possible	[55,59]
PEGylated liposomes (DPPC, DSPE-PEG2000)	Adrenaline	Hours, in vitro		[60]
Combination of fast and slow release liposomes (DSPC, DLPC, DMPC, DSPC)	Vaccine	Not specified		[61]
Small unilamellar liposomes (DSPC)	D-glucosamine sulphate, lubrication effect of phospholipids	Weeks, in vitro, days in vivo		[62]
Conventional and stealth liposomes (DPPG, DPPE-PEG2000)	Bevacizumab	Months in vitro, weeks in vivo		[63]
**Multivesicular liposomes**				
MVLs, DepoFoam^®^ (EPC, SPC, HSPC, DEPC, DOPC, DPPG)	Bupivacaine (Exparel^®^), ropivacaine, oleanolic acid, fluocinolone acetonide in cyclodextrin liraglutide, bevacizumab	Days, in vitro and in vivo	+ Hydrophilic and lipophilic drugs (but mostly hydrophilic drugs), biomacromolecule + Multiple aqueous compartments allow high encapsulation efficiency for hydrophilic drugs and slow release, avoiding a burst effec + Size in micron range prevents fast lymphatic uptak – Expensive production process including several steps, organic solvents and aseptic condition – Encapsulation of lipophilic drugs might alter multicompartment structure and not intensively investigated	[58,64,65,66,67,68,69,70,71,72]
**Liposomes in gel, coated liposomes**				
Liposomes in Pluronic^®^ F127 hydrogel (HPC, DPPG, DSPE-PEG2000)	Insulin, flurbiprofen, doxorubicin, paclitaxel	Days to weeks, in vivo	+ Hydrophilic and lipophilic drug + Embedding in matrix retains system at site of injection and could reduce lymphatic uptake + Surface coating could offer more possibilities for targeting and prolong drug releas – Further preparation steps require – Addition of more excipients with possible lower biocompatibility than phospholipid – Increase of viscosity could lead to worse injectability	[73,74,75]
Liposomes in PLGA-PEG-PLGA hydrogel (SPC)	Isoniazid, doxorubicin	Days, in vitro and in vivo		[76,77]
Liposomes in chitosan hydrogel (SPC, DPPC)	Doxorubicin, Carboxy-fluorescein	Days, in vitro and in vivo or not specified		[57,78]
Hybrid liposomes with chitosan (S75)	Curcumin	Days, in vitro		[79]
Hyaluronic acid-coated liposomes (DPPC, DOTAP)	Paclitaxel	Hours, in vitro		[80]
Ternary drug-cyclodextrin-liposomes complex (EPC)	Ropivacaine	Hours in vitro and in vivo		[81]
Capsosomes (EPC, DOTAP)	None	Not specified		[56]

* Abbreviations: EPC: L-α-phosphatidylcholine (egg), DEPC: 1,2-dierucoyl-sn-glycero-3-phosphocholine, DLPC: 1,2-dilauroyl-sn-glycero-3-phosphocholine, DMPC: 1,2-dimyristoyl-sn-glycero-3-phosphocholine, DOPA: 1,2-dioleoyl-sn-glycero-3-phosphate, DOPC: 1,2-dioleoyl-sn-glycero-3-phosphocholine, DOTAP: 1,2- dioleoyl-3-trimethylammonium-propan, DPPA: 1,2-dipalmitoyl-sn-glycero-3-phosphate, DPPC: 1,2-dipalmitoyl-sn-glycero-3-phosphocholine, DPPE-PEG2000: 1,2-dipalmitoyl-sn- glycero-3-phosphoethanolamine-N-[methoxy(polyethylene glycol)- 2000] (ammonium salt), DPPG: 1,2-dipalmitoyl-sn-gly- cero-3-phospho-(1′-rac-glycerol) (sodium salt), DSPC: 1,2-distearoyl-sn-glycero-3-phosphocholine, DSPE-PEG2000: 1,2-dis- tearoyl-sn-glycerol-3-phosphoethanolamine-N-[methoxy(poly-ethyleneglycol)-2000], DSPG: 1,2-distearoyl-sn-glycero-3-phospho-(1′-rac-glycerol), HPC: L-α-phosphatidylcholine, hydrogenated, HSPC: hydrogenated soy phosphatidylcholine SPC: L-α-phosphatidylcholine (soy), S75: soybean lecithin at 71% of phosphatidylcholine.

**Table 4:** Overview of semisolid and in situ forming drug delivery systems.

Principle	Encapsulated Drug	Release Time Range	Advantages (+) and Disadvantages (–)	Ref.
**Phospholipid-based phase separation gel (PPSG)**	Ropivacaine, pitavastatin, 5-fluorouracil, paclitaxel, doxorubicin, bromo-tetrandrine, dabigatran etexilate, exenatide, leuprolide, octreotide, insulin, incomplete Freund’s adjuvant	Days to weeks, in vitro and in vivo	+ Hydrophilic and lipophilic drugs, biomacromolecules, but – Drug needs to be soluble in ethanol + Fast and easy preparation and upscale possibilities + Low viscosity of precursor solution for easy injection + No initial burst release – Use of ethanol could cause side effects	[95,100,101,102,103,104,105,106,107,108,109,110]
**Vesicular phospholipid gels**	Interferon-beta-1b, granulocyte-colony stimulating factor, exenatide, FKBP51 antagonist	Days to weeks in vitro and in vivo	+ Hydrophilic and lipophilic drugs, biomacromolecules + Solvent-free manufacturing + Long-term storage form for liposomes – Semisolid behavior, high forces for injection required – Sterilization problematic, aseptic production required	[111,112,113,114,115]
**Phospholipid gel depo (PG Depot™)**	Small molecules, proteins, peptides	Days	+ Hydrophilic and lipophilic drugs, biomacromolecules + Low viscosity even with high phospholipid amount + Aqueous system with optional removal of water – Detailed information is patent-protected	[116,117]
**Liquid crystalline phases (cubosomes, hexosomes), FluidCrystal^®^, hybrid systems**	Bufalin, bupivacaine, minocycline, indomethacin, finasteride, doxorubicin, buprenorphine (Buvidal^®^, Brixadi^®^), sinomenine, docetaxel, leuprolide, octreotide, setmelanotide, Huperzine A, VEGF siRNA (combined with polyethylene-imine),	Days to weeks in vitro and in vivo	+ Hydrophilic and lipophilic drugs, biomacromolecules, but – Size of water channels need to be altered for release of macromolecules – High water content could induce burst release of hydrophilic drugs – High viscosity of cubic and hexagonal phase requires high injection forces, but + Dilution to cubosomes and hexosomes or use of water-free precursor solutions decrease viscosity + Choice of (phospho)lipids can control final properties – Contact with body fluids could change crystal structure, but + Precursor solutions use the transformation to liquid crystal systems upon contact with body fluids – Small changes in production can lead to different crystal structures – Highly controlled production process required	[98,118,119,120,121,122,123,124,125,126,127,128,129,130,131,132]
**Nanoemulsion**	Bupivacaine	Hours, in vitro and in vivo	+ Hydrophilic and lipophilic drugs + Fast and easy preparation – Addition of polymeric surfactants to stabilize the system could induce toxicity – Depot effect rather small	[133]
**Organogel**	Candesartan cilexetil, risperidone	Days in vitro and in vivo	– Only lipophilic drugs possible + Easy preparation – High viscosity complicates injection – Use of organogelators could increase toxicity	[134,135]

**Table 5:** Overview of depot systems based on solid particles and implants.

Principle	Encapsulated Drug	Release Time Range	Advantages (+) and Disadvantages (–)	Ref.
**Implants**	Ovalbumin, monoclonal antibody, ranibizumab, insulin, tumor peptide TRP2	Weeks in vitro	+ More versatile for lipophilic drugs and biomacromolecules + No drug leakage during storage + No induced pH change for enhanced protein stability – Injection with large needle diameter or surgery – Non-biodegradable systems have to be removed with surgery – Foreign body reactions around monolithic system	[151,156,157,158,159,160]
**Nanostructured lipid carriers**	Ondansetron	Days in vitro and in vivo	+ Hydrophilic and lipophilic drugs + High stability with no crystallization of the lipid – Use of organic solvents for preparation – Relatively fast release due to small size and resulting high surface	[161]
**Core-shell systems of lipid nano-/microparticles, combinations with polymers**	Ropivacaine, bupivacaine, risperidone, monoclonal antibody, lysozyme, insulin	Days to weeks in vitro and in vivo	+ Hydrophilic and lipophilic drugs, biomacromolecules + Prolonged release due to core–shell structure or embedding in gel matrix – Use of polymers with possible lower biocompatibility – More steps during preparation required	[154,155,162,163,164,165]
**Lipospheres or nanostructured lipid carriers in hydrogel**	Quetiapine, estradiol valerate	Hours to days in vitro and in vivo		[166,167]
